# Editorial: The effects of music on cognition and action, volume II

**DOI:** 10.3389/fnhum.2025.1557542

**Published:** 2025-01-29

**Authors:** Franco Delogu, Riccardo Brunetti, Cunmei Jang, Marta Olivetti Belardinelli

**Affiliations:** ^1^Department of Humanities, Social Sciences and Communication, Lawrence Technological University, Southfield, MI, United States; ^2^Experimental and Applied Psychology Laboratory, Department of Human Sciences, Università Europea di Roma, Rome, Italy; ^3^Music College, Shanghai Normal University, Shanghai, China; ^4^ECONA (Interuniversity Centre for the Research on Natural and Artificial Systems), Rome, Italy; ^5^Department of Psychology, Sapienza University of Rome, Rome, Italy

**Keywords:** gamma music, time perception in percussionists, tempo, music in preterm infants, music in cognitive neuromodulation, transfer effects, auditory steady state responses, monaural beats

Music, while apparently not essential for human survival, profoundly impacts neural processes, cognitive functions, and motor behavior through both passive and active engagement. This second volume of the Research Topic “*The effects of music on cognition and action*” builds on the 2021 Research Topic Volume I, presents contemporary research on the multifaceted influence of music on human cognition and action. These findings offer insights that can be categorized in the following four main themes.

## The effects of music on brain electrophysiology

Music's impact on cognition is rooted in its neurophysiological effects which are often measured with electrophysiological methods, like EEG. An emerging topic in cognitive neuroscience is the study of gamma oscillations, which are a brain wave patterns occurring in the frequency range of 20–150 Hz. A specific aspect of research linking gamma oscillations to cognitive enhancement and sensory integration (Herrmann et al., 2016) has been expanded by Yokota et al., who introduced the concept of “gamma music.” This innovation demonstrated the potential of gamma auditory steady-state responses (ASSRs) to modulate neural rhythms, opening avenues for cognitive and therapeutic applications. Similarly, the work of Chang et al. examined monaural beats combined with music, showing their capacity to induce theta brain activity and promote relaxation akin to sauna-induced states. These findings align with earlier studies on binaural beats' effects on cognition and mood (Chaieb et al., 2015), highlighting the exciting potential of auditory stimuli for non-invasive cognitive modulation.

## The effects of music on motor processes and metacognition

The relationship between music and motor processes remains a dynamic field of inquiry. Research from the early 21st century established music's ability to enhance motor coordination and flow states (Karageorghis and Priest, 2012). Expanding on this, Zhang et al. showed that fast-tempo music improves movement flow during brisk walking, correlating with specific neural activity. Li et al. explored musical feedback training's effects on metacognition, finding significant improvements on the self-awareness of thought processes and regulation despite inconclusive results on self-directed learning.

## The effects of music on neuroplasticity and cognitive enrichment

A recurring theme across this volume is music's ability to drive neuroplastic changes. From gamma oscillations to metacognitive gains, the research underscores music as a tool for cognitive enrichment. Building on earlier studies of musical skill and neural plasticity (Zatorre et al., 2007; Hallam, 2010; Herholz and Zatorre, 2012), Liao et al. revealed efficient synchronization processes in skilled percussionists, emphasizing how musical expertise shapes neural responses and motor control. Mittal et al. further examined temporal perception, uncovering nuanced modality-specific differences between musically trained and untrained individuals. These findings contribute to the always growing body of evidence supporting music's transferable benefits (Moreno and Bidelman, 2014), in cognitive and motor skills (Schellenberg, 2005), educational outcomes (Jaschke et al., 2018) and in second language acquisition (Marie et al., 2011; Delogu and Zheng, 2020; Zhang et al., 2024).

## Applications of music in therapy

Music's therapeutic potential is increasingly acknowledged in clinical contexts. Within our Research Topic, Kobus et al. showed music therapy's efficacy in improving preterm infants' vital signs and behavioral states, building on previous research in neonatal care (Loewy et al., 2013). Arnold et al. explored music's psychophysiological effects on pain perception, linking its therapeutic impact to both central and peripheral mechanisms. These findings reinforce earlier studies on music's role in pain management (Cepeda et al., 2006). Additionally, the trend toward personalized music-based interventions—emphasized by Chang's and Kobus' teams aligns with advancements in precision medicine, showcasing music's adaptability as a therapeutic tool (Thaut and Hoemberg, 2014).

## Future directions and challenges

Future research on the effects of music on cognition and action offers promising opportunities and has all the potential for the attainment of groundbreaking discoveries. Strengths include its interdisciplinary nature and wide-ranging impacts on cognition and health. Limitations involve standardization difficulties and ecological validity issues. As the mechanisms underlying music-induced cognitive and neural changes are complex and context-dependent, it is still a matter of debate whether the “transfer effects” of music on non-musical domains can be far reaching, with generalization of music skills to significantly different domains (Bigand and Tillmann, 2022) or just near-reaching cognitive functions closely related to music processing (Sala and Gobet, 2017). Future directions should focus on developing sophisticated methodologies bridging lab findings with real-world applications, addressing replicability, and integrating computational approaches. Larger sample sizes and open data practices will be crucial to strengthen evidence-based research on music-brain interactions and interventions. Long-term impacts and practical applications of interventions like gamma music require further study. Additionally, understanding individual differences in musical training and preferences is essential for optimizing interventions. Expanding cross-cultural studies can shed light on how diverse musical traditions influence cognition and emotion, helping identify universal vs. culture-specific effects (Trehub et al., 2015).

## Conclusion

Our Research Topic of studies highlights some of the most relevant effects of music on cognition and action, as observed in multiple aspects of brain function and behavior. The studies presented in this volume significantly advance our understanding of music's profound effects on brain states, behavior, and motor processes. Highlighting four macro-themes, this Research Topic of studies positions music as a uniquely versatile tool for enhancing cognitive, emotional, and physical wellbeing. As research evolves, it promises to unlock new opportunities for leveraging music's power across diverse life domains and cultural contexts.
